# Withaferin A Induces Oxidative Stress-Mediated Apoptosis and DNA Damage in Oral Cancer Cells

**DOI:** 10.3389/fphys.2017.00634

**Published:** 2017-09-07

**Authors:** Hsueh-Wei Chang, Ruei-Nian Li, Hui-Ru Wang, Jing-Ru Liu, Jen-Yang Tang, Hurng-Wern Huang, Yu-Hsuan Chan, Ching-Yu Yen

**Affiliations:** ^1^Department of Biomedical Science and Environmental Biology, Kaohsiung Medical University Kaohsiung, Taiwan; ^2^Department of Medical Research, Kaohsiung Medical University Hospital Kaohsiung, Taiwan; ^3^Cancer Center, Kaohsiung Medical University Hospital; Kaohsiung Medical University Kaohsiung, Taiwan; ^4^Research Center for Natural Products and Drug Development, Kaohsiung Medical University Kaohsiung, Taiwan; ^5^Institute of Medical Science and Technology, National Sun Yat-Sen University Kaohsiung, Taiwan; ^6^Institute of Biomedical Science, National Sun Yat-Sen University Kaohsiung, Taiwan; ^7^Department of Radiation Oncology, Faculty of Medicine, College of Medicine, Kaohsiung Medical University Kaohsiung, Taiwan; ^8^Department of Radiation Oncology, Kaohsiung Medical University Hospital Kaohsiung, Taiwan; ^9^Department of Radiation Oncology, Kaohsiung Municipal Ta-Tung Hospital Kaohsiung, Taiwan; ^10^Department of Oral and Maxillofacial Surgery Chi-Mei Medical Center Tainan, Taiwan; ^11^School of Dentistry, Taipei Medical University Taipei, Taiwan

**Keywords:** oral cancer, selective killing, apoptosis, oxidative stress, withaferin A, *N*-acetylcysteine

## Abstract

Withaferin A (WFA) is one of the most active steroidal lactones with reactive oxygen species (ROS) modulating effects against several types of cancer. ROS regulation involves selective killing. However, the anticancer and selective killing effects of WFA against oral cancer cells remain unclear. We evaluated whether the killing ability of WFA is selective, and we explored its mechanism against oral cancer cells. An MTS tetrazolium cell proliferation assay confirmed that WFA selectively killed two oral cancer cells (Ca9-22 and CAL 27) rather than normal oral cells (HGF-1). WFA also induced apoptosis of Ca9-22 cells, which was measured by flow cytometry for subG1 percentage, annexin V expression, and pan-caspase activity, as well as western blotting for caspases 1, 8, and 9 activations. Flow cytometry analysis shows that WFA-treated Ca9-22 oral cancer cells induced G2/M cell cycle arrest, ROS production, mitochondrial membrane depolarization, and phosphorylated histone H2A.X (γH2AX)-based DNA damage. Moreover, pretreating Ca9-22 cells with *N*-acetylcysteine (NAC) rescued WFA-induced selective killing, apoptosis, G2/M arrest, oxidative stress, and DNA damage. We conclude that WFA induced oxidative stress-mediated selective killing of oral cancer cells.

## Introduction

Most anticancer drugs effectively kill cancer cells; however, they also non-selectively kill normal cells, which limits their therapeutic value. It is currently believed that deregulated cell proliferation and deregulated apoptosis contributes to carcinogenesis (Reed, 1999). When the balance between proliferation and apoptosis is interrupted during tumor development, cell proliferation is deregulated (Scully et al., 2000; Evan and Vousden, 2001). Therapeutics that target oral squamous cell carcinoma (OSCC) cell proliferation and apoptosis regulators can enable these cancer cells to evade the regulatory system (Evan and Vousden, 2001).

Reactive oxygen species (ROS) are primarily generated in the mitochondria, and they cause a loss of mitochondrial membrane potential (MMP) (Li et al., 2006; Oh and Lim, 2006). ROS is an important inducer for the early stages of apoptosis (Samhan-Arias et al., 2004) and DNA damage (Barzilai and Yamamoto, 2004; Chen et al., 2016). Several drugs that modulate ROS have been reported (Nicco et al., 2005; Trachootham et al., 2006; Wu and Hua, 2007; Widodo et al., 2010) to regulate apoptosis for selective killing. Thus, ROS might mediate the selective activation of apoptosis for selective killing in cancer chemotherapy (Pollack et al., 2001; Daniel et al., 2003; Real et al., 2004).

Recently, several natural products have also been reported to induce apoptosis involving ROS (Ding et al., 2009; Chiu et al., 2013; Lee et al., 2013; Vyas and Singh, 2014). For example, the antiproliferation effect of cancer cells for the natural product from *Withania somnifera* (*W. somnifera*) is commonly reported to steroidal lactones (withanolides) (Vyas and Singh, 2014). Of the various withanolides derived from the root or leaf of *W. somnifera*, withaferin A (WFA) appears to be the most active against cancer (Vyas and Singh, 2014). The molecular anticancer activities of WFA have been reported through its antioxidant, anti-inflammatory and metabolic activities (Vanden Berghe et al., 2012; Vyas and Singh, 2014). WFA also induces ROS production and mitochondria-mediated caspase activation in apoptosis of HL-60 myeloid leukemia cells (Malik et al., 2007). Thus, WFA has potential for modulating apoptosis and oxidative stress. Moreover, WFA has been widely used in several types of cancer (Malik et al., 2007; Uma Devi et al., 2008; Woo et al., 2014; Lee et al., 2015), but its use in oral cancer cells has not been sufficiently investigated.

We therefore hypothesized that WFA killed oral cancer cells by regulating oxidative stress-mediated apoptosis. We tested this hypothesis by evaluating cell viability, cell cycle changes, annexin V, caspases, ROS, MMP, and DNA damage. Moreover, the role of oxidative stress in WFA-induced cell killing of oral cancer cells was examined by the addition of an antioxidant *N*-acetylcysteine (NAC). Therefore, this work sheds light on exploring the roles of oxidative stress-mediated WFA-induced cell killing mechanism of oral cancer cells.

## Materials and methods

### Cell cultures and chemicals

Ca9-22 and CAL 27 oral cancer cell lines and HGF-1 normal human gingival fibroblast cell lines were kept in Dulbecco's Modified Eagle's Medium (DMEM) (Gibco, Grand Island, NY, USA) plus 10% fetal bovine serum (FBS) at 37°C in a humidified atmosphere containing 5% CO_2_, as previously described (Chang Y. T. et al., 2016). WFA was purchased from Selleckchem.com (Houston, TX, USA) and dissolved in dimethyl sulfoxide (DMSO) for experiments. An antioxidant or free radical scavenger NAC (Sigma-Aldrich; St. Louis, MO, USA) was pretreated before WFA treatment to diminish cellular ROS and confirm the role of oxidative stress in WFA treatment.

### Cell viability

Cell viability was measured using an MTS assay (CellTiter 96 Aqueous One Solution; Promega, Madison, WI, USA), as previously described (Chiu et al., 2013).

### Cell cycle distribution

DNA content was detected using 7-aminoactinomycin D (7AAD), a DNA dye, (Vignon et al., 2013). After fixation in 70% ethanol, cells were treated with 1 μg/ml 7AAD for 30 min at 37°C. Finally, the cells were resuspended in phosphate-buffered saline (PBS), and signals were examined using the FL3 channel of the Accuri C6 flow cytometer, and software (BD Biosciences, Franklin Lakes, New Jersey, USA). The G2/M percentage was calculated as the G2/M population among the whole cell cycle phases (G1, S, and G2/M).

### Annexin V/DNA content assay to measure apoptosis

Apoptosis was measured using annexin V/propidum iodide (PI) (Strong Biotech Corp., Taipei, Taiwan; and Sigma-Aldrich) or annexin V/7AAD, as previously described (Chiu et al., 2011). After cells were treated with the WFA- or NAC/WFA (NAC pretreatment and WFA posttreatment), they were treated for 30 min at 37°C with annexin V-fluorescein isothiocyanate (annexin V-FITC) (10 μg/ml) and PI (5 μg/ml) or annexin V-FITC/7AAD (1 μg/ml), and then resuspended in PBS for analysis using the FL1/FL2 or FL1/FL3 channels of the Accuri C6 flow cytometer and software, respectively.

### Pan-caspase assay to measure apoptosis

Apoptosis was measured using a generic caspase activity assay kit (Fluorometric-Green: ab112130; Abcam, Cambridge, UK) (Yeh et al., 2012) for detecting the activity of caspases-1, 3-9. After the cells had been treated with WFA or NAC/WFA, they were treated with 2 μl of 500X TF2-VAD-FMK per 2 ml of medium for 2 h in a cell cultureincubator. After the cells had been washed with PBS, they were resuspended in 0.5 ml of assay buffer, and signals were immediately detected using the FL1 channel of the Accuri C6.

### Western blotting of caspase signaling to measure apoptosis

The detailed procedures of western blotting are described previously (Chen et al., 2016). Briefly, 30 μg of protein lysates was loaded for 10% SDS-PAGE (sodium dodecylsulfate polyacrylamide gel electrophoresis), and transferred to PVDF (polyvinylidene fluoride) membranes (Pall Corp., Port Washington, NY, USA). Protein blocking was done using 5% non-fat milk in Tris-buffered saline with Tween-20. Subsequently, the lysates were treated with primary antibodies: cleaved caspase-8 (Asp391) (18C8) rabbit monoclonal antibody (mAb); cleaved PARP [poly(ADP-ribose) polymerase] (Asp214) (D64E10) XP® rabbit mAb; cleaved caspase-3 (Asp175) (5A1E) rabbit mAb; and cleaved caspase-9 (Asp330) (D2D4) rabbit mAb (Cell Signaling Technology, Inc., Danvers, MA, USA) (diluted 1:1000). mAb-β-actin (clone AC-15) (#A5441; Sigma-Aldrich) (diluted 1:5,000) was used as a control. Their matched secondary antibodies were also used. Signal was detected using a substrate (WesternBright™ ECL HRP: #K-12045-D50; Advansta, Menlo Park, CA, USA). The densitometry quantification of blot was determined by ImageJ freeware.

### ROS production assay

2′,7′-Dichlorodihydrofluorescein diacetate (H_2_DCF-DA) was used for ROS detection, as previously described (Shih et al., 2014). After the cells were treated with WFA or NAC/WFA, they were treated with 100 nM of H_2_DCF-DA in PBS for 30 min in a cell culture incubator. After the cells were harvested and washed, they were resuspended in PBS and their signals were immediately analyzed using the FL1 channel of the Accuri C6.

### Mitochondrial membrane potential (MMP) assay

MMP was detected using an assay kit (MitoProbe™ 3,3′-diethyloxacarbocyanine iodide (DiOC_2_(3)) (Invitrogen, San Diego, CA, USA)) as previously described (Yen et al., 2012). After the cells were treated with WFA or NAC/WFA, they were washed with PBS and treated with 10 of 10 μM DiOC_2_(3) per 1 ml of medium/well in a 6-well plate in a cell culture incubator for 20–30 min. After the cells were harvested and washed, they were resuspended in PBS and their signals were immediately analyzed using the FL1 channel of the Accuri C6.

### Phosphorylated histone H2A.X (γH2AX) assay

γH2AX, DNA double-strand break biomarker, was detected using flow cytometry as previously described (Chen et al., 2016). After the cells were treated with WFA or NAC/WFA, they were fixed with 70% ethanol. After they had been washed, they were treated with 2 μg/ml of phospho-Histone 2A.X (Ser139) Antibody (H2AX) (sc-101696; Santa Cruz Biotechnology, Santa Cruz, CA, USA) at 4°C for 1 h. After the cells were washed, they were treated with a secondary antibody (Jackson Laboratory, Bar Harbor, ME, USA) at room temperature for 30 min. Finally, the cells were treated in 20 μg/ml of PI, and their signals were detected using the FL1/FL2 channels of the Accuri C.

### Statistical analysis

All data are presented as means ± SD. Each analysis was performed in three separate experiments at different times (*n* = 3). All data were analyzed using Student paired *t*-test of Sigmaplot 10.0 (Scientific Data Analysis and Graphing Software, Systat Software Inc., Chicago, IL, USA).

## Results

### The viability of oral cancer cells and normal oral cells treated with WFA was significantly affected in WFA-treated cells with NAC pretreatment

**Figure 1:**
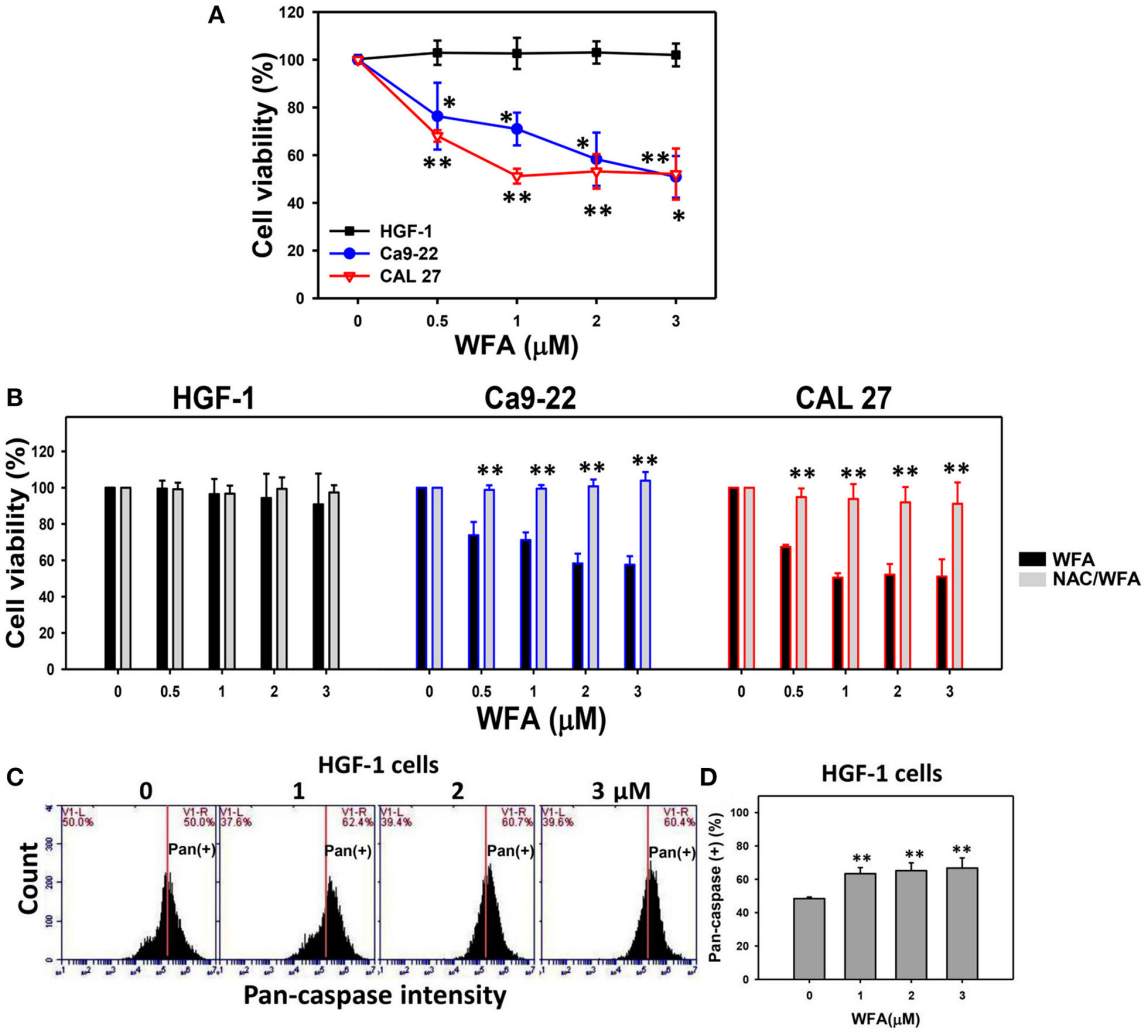
MTS-based cell viability of WFA-treated oral cancer cells and normal cells and its changes after NAC pretreatment. **(A)** Oral cancer cells (Ca9-22 and CAL 27) and oral normal cells (HGF-1) were treated with WFA (0–3 μM) for 24 h. **(B)** NAC pretreatment effect on MTS-based cell viability of WFA-treated oral cancer and oral normal cells. Cells were pretreated with 2 mM NAC for 1 h and post-treated with WFA (0–3 μM) for 24 h. **(C)** Typical patterns of pan-caspase activity for WFA-treated oral normal cells (HGF-1). Cells were treated with different concentrations (0–3 μM) of WFA for 24 h for pan-caspase analysis. V1-L and V1-R listed in the corner of each plot, respectively, indicate the percentages of cell population (black color) on the left and right sides of the vertical line. In the control, the percentages of cell populations for V1-L and V1-R are 50% each (Chang H. W. et al., 2016). The position of this line is at the same position for all treatments as the control setting. Positive (+) %, the cell population on the right side of the line, is indicated in each **(D)** Pan-caspase-based apoptosis (+) (%) for **(C)**. Data are means ± SDs (*n* = 3). **(A,D)** ^*^p < 0.05 and ^**^p < 0.001 against control (0 μM). **(B)** ^**^p < 0.001 for comparison between WFA and NAC/WFA (NAC pretreatment and WFA posttreatment).

MTS assays showed that the relative cell viability (%) of the Ca9-22 and CAL 27 oral cancer cells were significantly lower than control after 24 h WFA treatments of 0.5, 1, 2, and 3 μM in a dose-dependent manner (Figure 1A). In contrast, HGF-1 normal oral cells treated with WFA showed no reduction in viability.

The involvement of oxidative stress in drug treatment is usually validated by pretreating cells with an antioxidant like NAC (Chan et al., 2006; Shieh et al., 2014; Hung et al., 2015; Lien et al., 2017). Cells treated with NAC-only [NAC pretreatment (2 mM)/WFA posttreatment (0 μM)] differed non-significantly from untreated controls (no NAC pretreatment and no WFA posttreatment in all three types of cells (Figure 1B). Moreover, WFA-induced antiproliferation was significantly inhibited in two types of WFA-treated oral cancer cells with NAC pretreatment (NAC/WFA) (*p* < 0.05–0.001).

To further validate the low cytotoxicity of WFA-treated HGF-1 normal oral cells, the levels of WFA-induced apoptosis in HGF-1 cells were evaluated using the pan-caspase assay. The flow cytometric pan-caspase patterns of WFA-treated HGF-1 cells are shown in Figure 1C. Generic caspase activities in WFA-treated HGF-1 cells slightly increased at 1–3 μM WFA about 60% compared to the control (50%) (*p* < 0.001) (Figure 1D), suggesting that WFA only induced minor signs of apoptosis (only 10% induction) with low cytotoxicity to HGF-1 normal oral cells compared to the control.

### Cell cycle-perturbed distribution of CA9-22 oral cancer cells treated with WFA was inhibited in WFA-treated cells with NAC pretreatment

**Figure 2:**
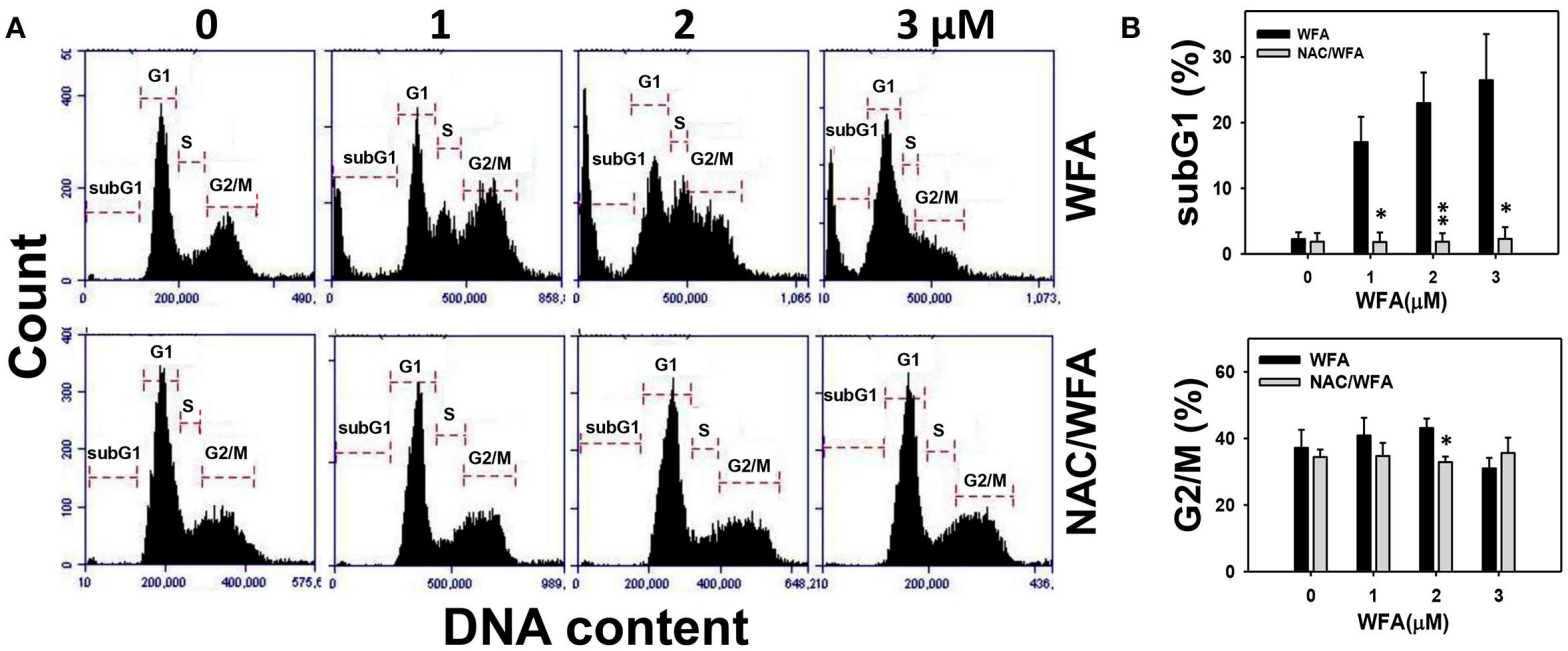
The cell cycle distribution of WFA-treated Ca9-22 oral cancer cells and its changes after NAC pretreatment. **(A)** Typical cell cycle patterns of WFA-treated Ca9-22 oral cancer cells with and without NAC pretreatment. With and without NAC pretreatment (2 mM NAC for 1 h), cells were post-treated with WFA (0–3 μM) for 24 h. **(B)** SubG1 and G2/M phases (%) for **(A)**. Data are means ± SDs (*n* = 3). ^*^p < 0.05 and ^**^p < 0.001 for comparison between WFA and NAC/WFA for each concentration of WFA. NAC/WFA, NAC pretreatment and WFA posttreatment.

The flow cytometric cell cycle patterns of Ca9-22 oral cancer cells treated with WFA are shown in Figure 2A (top panel). Sub-G1 populations were higher in Ca9-22 cells treated with WFA than the control (Figure 2B, top panel). The flow cytometric cell cycle patterns of WFA and NAC/WFA-treated Ca9-22 cells are shown in Figure 2A (bottom panel). WFA-induced sub-G1 accumulation (Figure 2B, top panel) was significantly inhibited in WFA-treated Ca9-22 cells with NAC pretreatment (NAC/WFA) (*p* < 0.001). Moreover, G2/M populations were higher in Ca9-22 cells treated with WFA ranging from 1 to 2 μM (Figure 2B, bottom panel). WFA-induced G2/M accumulation (Figure 2B, bottom panel) was significantly inhibited in WFA (2 μM)-treated Ca9-22 cells with NAC pretreatment (NAC/WFA) (*p* < 0.05).

### Annexin V/PI-induced apoptosis of CA9-22 oral cancer cells treated with WFA was inhibited in WFA-treated cells with NAC pretreatment

**Figure 3:**
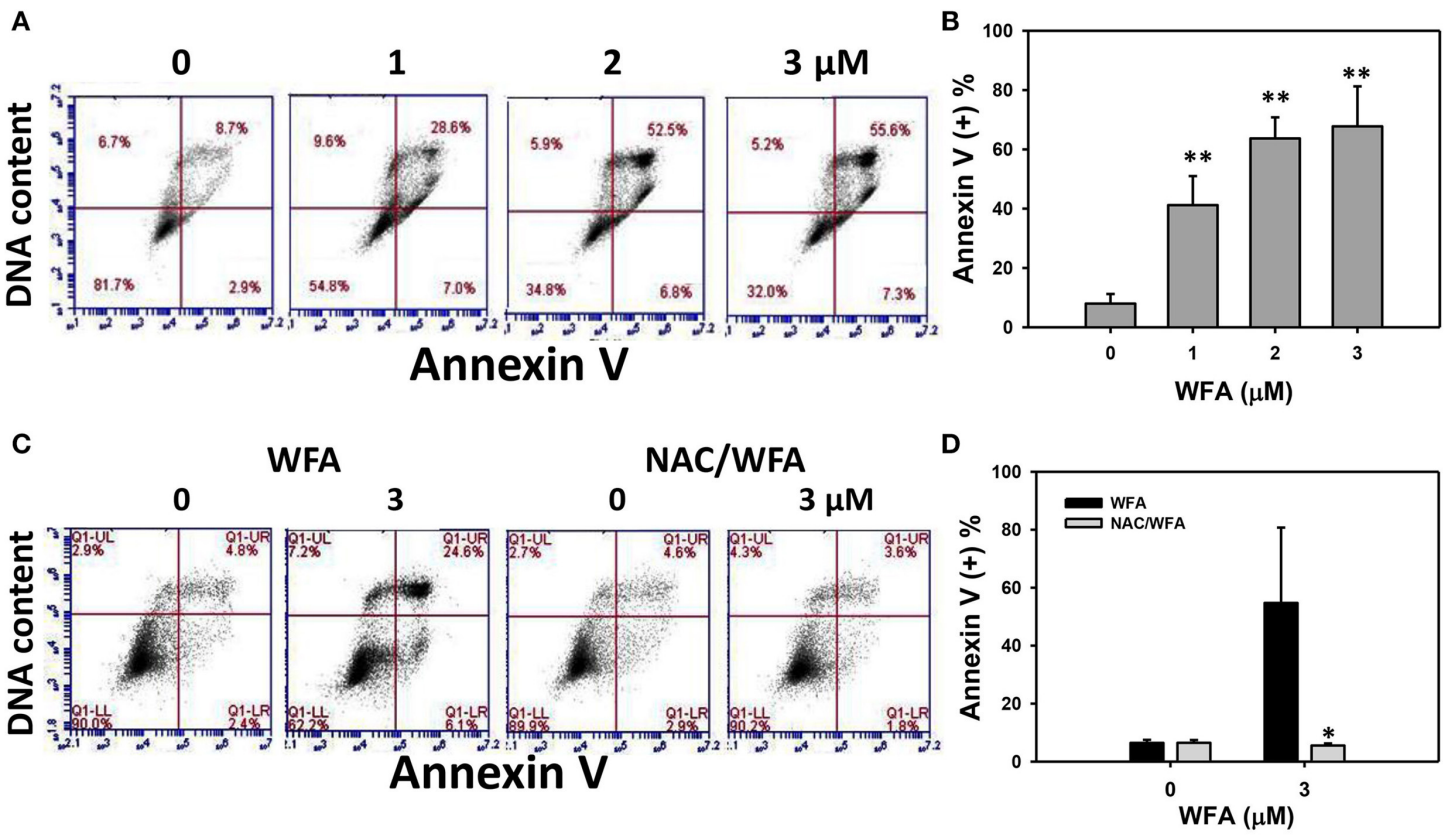
Apoptosis of WFA-treated Ca9-22 oral cancer cells and its changes after NAC pretreatment. **(A)** Typical patterns of annexin V/DNA content method for WFA-treated Ca9-22 oral cancer cells. Cells were treated with WFA (0–3 μM) of 24 h for flow cytometry analyses. **(B)** Annexin V positive (+) (%) for **(A)**. **(C)** Typical annexin/DNA content-based apoptosis patterns of NAC effect on WFA-treated Ca9-22 cells. With or without NAC pretreatment (2 mM NAC for 1 h), cells were post-treated with WFA (0 and 3 μM) for 24 h. **(D)** Annexin/DNA content-based apoptosis (+) (%) for **(C)**. Data are means ± SDs (*n* = 3). **(B)** ^**^p < 0.001 against control (0 μM). **(D)** ^*^p < 0.05 for comparison between WFA and NAC/WFA (NAC pretreatment and WFA posttreatment).

The flow cytometric annexin V/PI patterns of Ca9-22 oral cancer cells treated with WFA are shown in Figure 3A. The annexin V positive (+) expression (%) for WFA-treated Ca9-22 cells was higher than the control in a dose-dependent manner (Figure 3B).

The flow cytometric annexin V/PI patterns of WFA- and NAC/WFA-treated Ca9-22 cells are shown in Figure 3C. Annexin V (+) expression in cells treated with NAC differed non-significantly from those in untreated controls of WFA-treated Ca9-22 cells (Figure 3D, left). Moreover, WFA-induced annexin V-based apoptosis was significantly inhibited in WFA-treated Ca9-22 cells with NAC pretreatment (NAC/WFA) (Figure 3D, right) (*p* < 0.001).

### Pan-caspase-based apoptosis of CA9-22 oral cancer cells treated with WFA was inhibited in WFA-treated cells with NAC pretreatment

**Figure 4:**
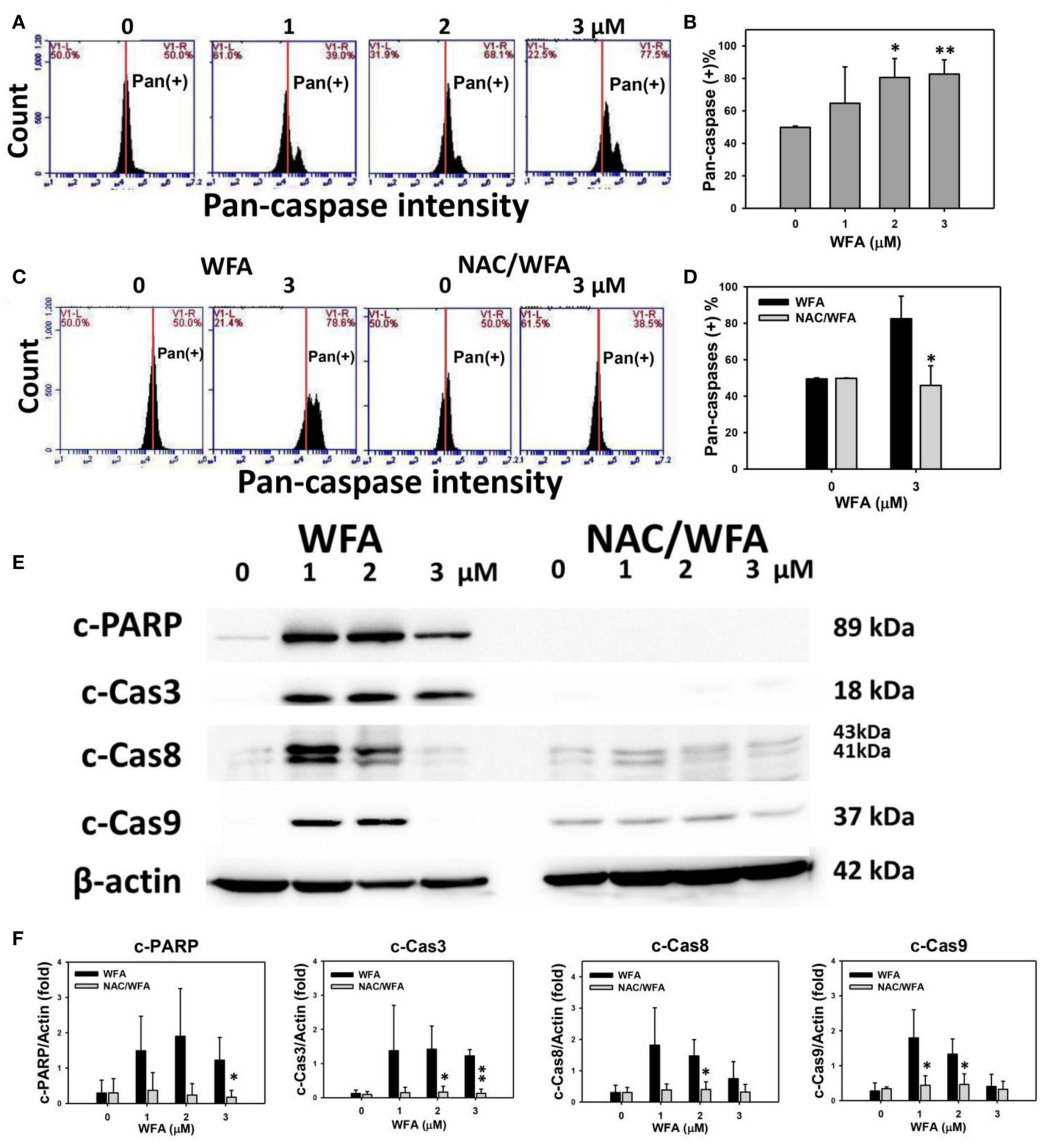
Caspase activity in WFA-treated Ca9-22 oral cancer cells and its changes after NAC pretreatment. **(A)** Typical patterns of pan-caspase activity for WFA-treated oral cancer Ca9-22 cells. Cells were treated with different concentrations (0–3 μM) of WFA for 24 h for pan-caspase analysis. V1-L and V1-R labeled in the corner of each plot respectively indicate the percentages of cell population (black color) in the left and right sides of the vertical line. In control, the percentages of cell population for V1-L and V1-R are 50% each (Chang H. W. et al., 2016). The position of this line is at the same position for all treatments as the control setting. Positive (+) %, the cell population in the right side of the line, is indicated in each panel. **(B)** Pan-caspase-based apoptosis (+) (%) for **(A)**. **(C)** Typical pan-caspase-based apoptosis patterns of NAC effect on WFA-treated Ca9-22 cells. Cells were pretreated with 2 mM NAC for 1 h and post-treated with WFA (0–3 μM) for 24 h. **(D)** Pan-caspase-based apoptosis (+) (%) for **(C)**. **(E)** Expressions of apoptosis signaling proteins (cleaved caspases 3, 8, 9, and PARP) in Ca9-22 cells with WFA treatment with or without NAC pretreatment. Cells were pretreated with 2 mM NAC for 1 h and post-treated with WFA (0, 1, and 3 μM) for 24 h. β-actin was an internal control. Western blotting for WFA and NAC/WAF was performed in the same gel with the same exposure time. NAC/WFA, NAC pretreatment, and WFA posttreatment. **(F)** Statistic result for three separate western blottings in **(E)**. The densitometry quantification of blot was determined by ImageJ freeware. Data are means ± SDs (*n* = 3). **(B)** ^*^p < 0.05 and ^**^p < 0.001 against control (0 μM). **(D,F)** ^*^p < 0.05 and ^**^p < 0.001 for comparison between WFA and NAC/WFA.

The involvement of caspases in the apoptosis of WFA-treated Ca9-22 cells was examined using a TF2-VAD-FMK flow cytometric assay (Figure 4). The flow cytometric pan-caspase patterns of WFA-treated Ca9-22 cells are shown in Figure 4A. Generic caspase activities in Ca9-22 cells treated with WFA ranging from 2 to 3 μM showed a significant increase above 80% compared to the control (50%) (*p* < 0.05–0.001), i.e., 30% induction (Figure 4B). The flow cytometric pan-caspase patterns of WFA- and NAC/WFA-treated Ca9-22 cells are provided in Figure 4C. In Figure 4D, the WFA-induced pan-caspase-based apoptosis in Ca9-22 cells was significantly inhibited in WFA-treated Ca9-22 cells with NAC pretreatment (NAC/WFA) (*p* < 0.05).

The involvement of caspases in the change of pan-caspase activity was validated using western blotting. In Figures 4E,F, the expression of apoptosis signaling proteins, such as cleaved PARP and cleaved-caspases 3, 8, and 9, was higher in WFA-treated Ca9-22 cells; although some of them declined at a concentration of 3 μM. In contrast, these caspase signaling proteins of the WFA-treated Ca9-22 cells were inhibited in WFA-treated Ca9-22 cells with NAC pretreatment (NAC/WFA).

### ROS generated oxidative stress of CA9-22 oral cancer cells treated with WFA was inhibited in WFA-treated cells with NAC pretreatment

**Figure 5:**
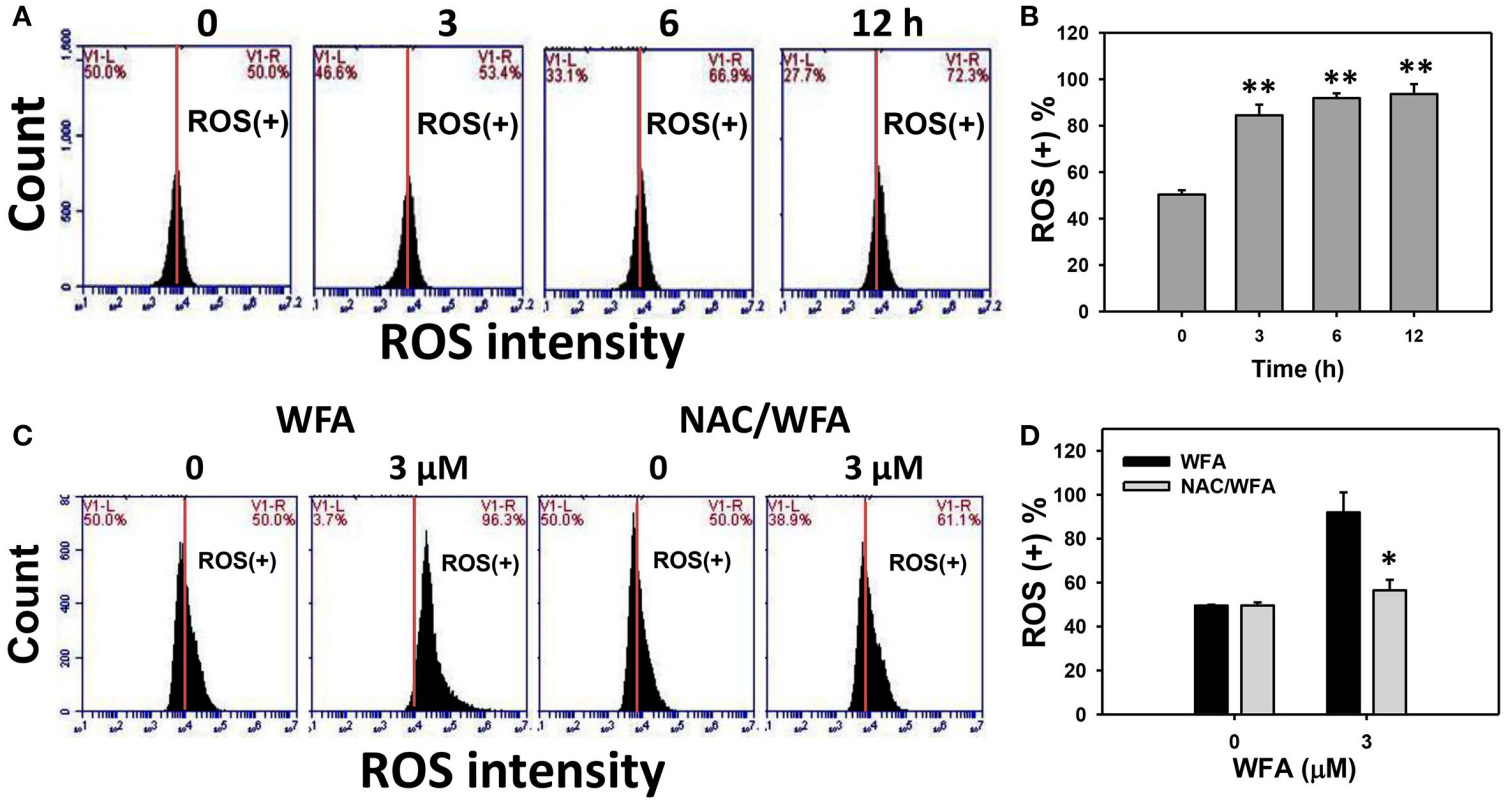
ROS levels of WFA-treated Ca9-22 oral cancer cells and its changes after NAC pretreatment. **(A)** Typical ROS patterns of WFA-treated Ca9-22 oral cancer cells. Cells were treated with WFA (0–3 μM) for 3, 6, and 12 h for flow cytometry analysis. V1-L and V1-R labeled in the corner of each plot respectively indicate the percentages of cell population (black color) in the left and right sides of the vertical line. In the control, the percentages of cell populations for V1-L, and V1-R are 50% each (Chang H. W. et al., 2016). The position of this line is at the same position for all treatments as the control setting. Positive (+) %, the cell population in the right side of the line, is indicated in each panel. **(B)** ROS (+) intensity (%) for **(A)**. **(C)** Typical ROS patterns of NAC effect on WFA-treated Ca9-22 cells. Cells were pretreated with 2 mM NAC for 1 h and post-treated with WFA (0–3 μM) for **(24 h)**. **(D)** ROS (+) intensity (%) for **(C)**. NAC/WFA, NAC pretreatment and WFA posttreatment. Data are means ± SDs (*n* = 3). **(B)** ^**^p < 0.001 against control (0 μM). **(D)** ^*^p < 0.05 for comparison between WFA and NAC/WFA for each concentration of WFA.

The ROS positive (+) patterns of WFA-treated Ca9-22 cells for 3, 6, and 12 h are displayed in Figure 5A. The ROS (+) (%) expression of WFA-treated Ca9-22 cells was significantly and time-dependently higher (*p* < 0.001) (Figure 5B).

Figure 5C shows the ROS (+) patterns of WFA- and NAC/WFA-treated Ca9-22 cells at 24 h of WFA treatment. Figure 5D shows higher ROS (+) expression in Ca9-22 cells after WFA treatment when compared to the control in a dose-dependent manner. ROS production induced by WFA treatment was significantly inhibited in WFA-treated Ca9-22 cells with NAC pretreatment (NAC/WFA) (Figure 5D) (*p* < 0.001).

### Mitochondrial membrane potential (MMP) of CA9-22 oral cancer cells treated with WFA was inhibited in WFA-treated cells with NAC pretreatment

**Figure 6:**
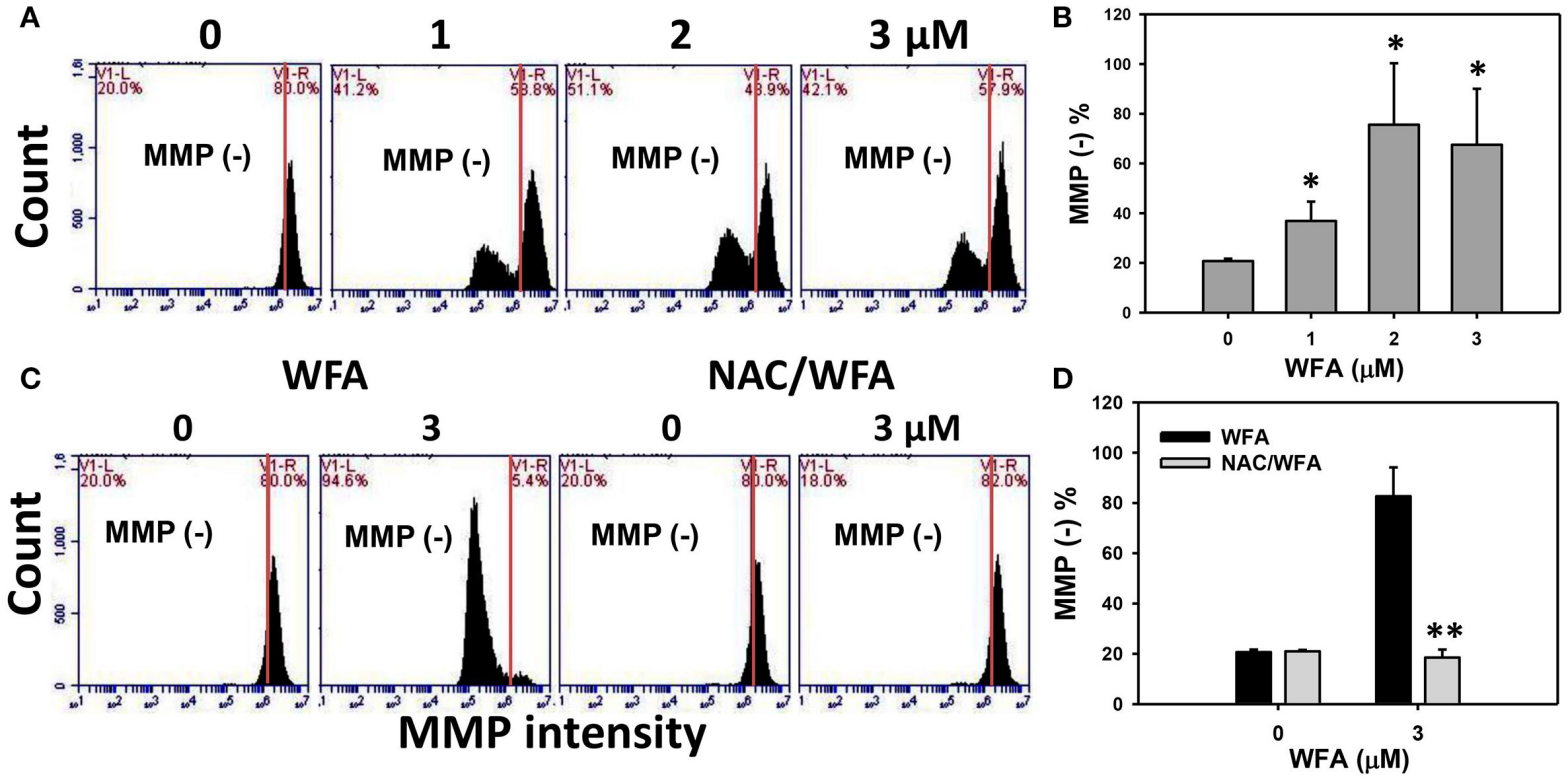
MMP levels of WFA-treated Ca9-22 oral cancer cells and its changes after NAC pretreatment. **(A)** Typical MMP patterns of WFA-treated Ca9-22 oral cancer cells. Cells were treated with WFA (0–3 μM) for 24 h for flow cytometry analysis. V1-L and V1-R labeled in the corner of each plot respectively indicate the percentages of cell populations (black color) in the left and right sides of the vertical line. In control, the percentages of cell populations for V1-L and V1-R are 20 and 80%, respectively (Chang H. W. et al., 2016). The position of this line is at the same position for all treatments as the control setting. MMP-negative (−) (%), the cell population in the left side of the line, is indicated in each panel. **(B)** MMP (−) intensity (%) for **(A)**. **(C)** Typical MMP patterns of the effect of NAC on WFA-treated Ca9-22 cells. Cells were pretreated with 2 mM NAC for 1 h and post-treated with WFA (0–3 μM) for 24 h. **(D)** MMP (−) (%) for **(C)**. Data are means ± SDs (*n* = 3). **(B)** ^*^p < 0.05 against control (0 μM). **(D)** ^**^p < 0.001 for comparison between WFA and NAC/WFA (NAC pretreatment and WFA posttreatment).

The MMP negative (−) expression (%) of WFA-treated Ca9-22 is displayed in Figure 6A. After Ca9-22 cells were treated with WFA for 24 h, the MMP (−) expression (%) of WFA-treated Ca9-22 cells were increased (Figure 6B), suggesting that WFA induces mitochondrial membrane depolarization in Ca9-22 cells.

The flow cytometric MMP patterns of WFA- and NAC/WFA-treated Ca9-22 cells are displayed in Figure 6C. WFA-induced increase of MMP (−) expression was significantly inhibited in WFA-treated Ca9-22 cells with NAC pretreatment (NAC/WFA) (*p* < 0.001) (Figure 6D).

### γH2AX-based DNA damage of CA9-22 oral cancer cells treated with WFA was inhibited in WFA-treated cells with NAC pretreatment

**Figure 7:**
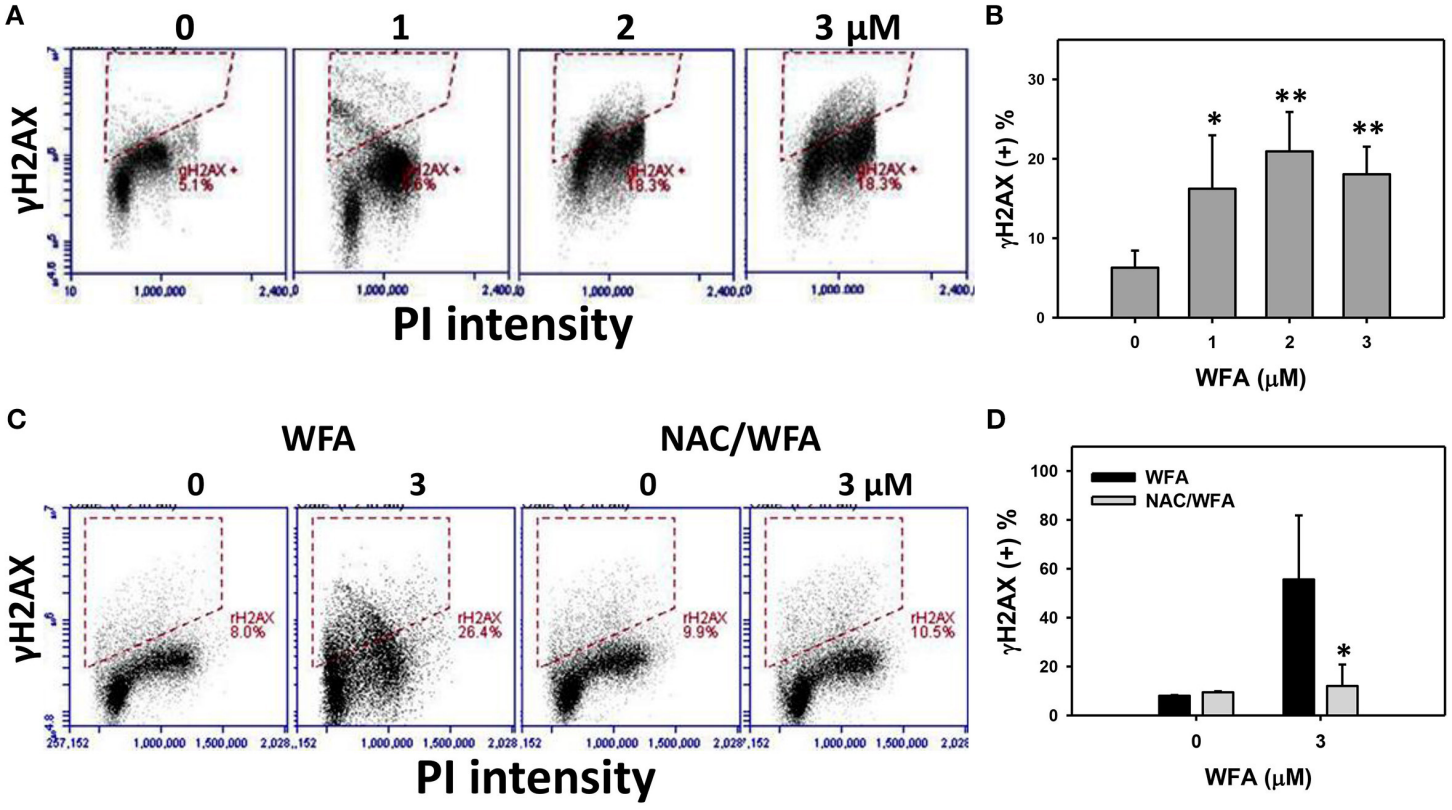
DNA double strand breaks of WFA-treated Ca9-22 oral cancer cells and its changes after NAC pretreatment. **(A)** Ca9-22 oral cancer cells treated with different concentrations (0-3 μM) of WFA for 24 h were stained with γH2AX. **(B)** γH2AX positive (+) intensity (%) for **(A)**. **(C)** Typical DNA damage patterns of NAC effect on WFA-treated Ca9-22 cells. Cells were pretreated with 2 mM NAC for 1 h and post-treated with WFA (0–3 μM) for 12 h. **(D)** γH2AX (+) intensity (%) for **(C)**. Data are means ± SDs (*n* = 3). **(B)** ^*^p < 0.05 and ^**^p < 0.001 against control (0 μM). **(D)** ^*^p < 0.05 for comparison between WFA and NAC/WFA (NAC pretreatment and WFA posttreatment).

γH2AX was flow cytometrically measured the DNA damage in WFA-treated Ca9-22 oral cancer cells. The percentages and profiles of γH2AX positive (+) stained cells were shown for the 24 h treatments with 0, 1, 2, and 3 μM of WFA (Figure 7A). After 24 h of WFA treatment, the % of γH2AX (+) stained cells was significantly higher than the control (*p* < 0.05–0.001) (Figure 7B).

The flow-cytometry γH2AX patterns of WFA- and NAC/WFA-treated Ca9-22 cells were provided in Figure 7C. WFA-induced γH2AX expression was significantly inhibited in WFA-treated Ca9-22 cells with NAC pretreatment (NAC/WFA) (*p* < 0.05) (Figure 7D).

## Discussion

WFA has anticancer (Uma Devi et al., 2008; Woo et al., 2014; Lee et al., 2015) and ROS production (Lee et al., 2009; Li et al., 2017; Liu et al., 2017) effects in several types of cancer. However, the selective killing effect of WFA was rarely investigated as yet, especially in oral cancer cells. The aim of this study is to examine the selective killing effects of WFA against oral cancer cells and to explore the role of oxidative stress in WFA-treated oral cancer cells. The cell killing, apoptosis, cell cycle, DNA damage, and oxidative stress effects of WFA in oral cancer cells are discussed as follows.

### Comparison of IC_50_ using WFA in different types of cancer cells

Different cancer cell types are sensitive to WFA. For example, the IC_50_ values of WFA were 2 μM for myeloid leukemia (HL-60) cells (24 h; MTT assay) (Malik et al., 2007), about 2 μM for pancreatic cancer cells (Panc-1 and MIAPaCa-2) (48 h; MTS assay) (Li et al., 2015), and 0.2–1.2 μM for cervical cancer cells (C33a, CaSki, HeLa, and SiHa) (24 h; MTT assay) (Munagala et al., 2011). However, the selective killing effect of WFA was not thoroughly investigated in these studies. We also found that the IC_50_-values of WFA in Ca9-22 and CAL 27 oral cancer cells were 3 and 2 μM (24 h; MTS assay), respectively. In contrast, WFA did not harm HGF-1 normal oral cells. Accordingly, WFA induces selective killing against oral cancer cells without adverse effects for the viability of normal oral cells. Therefore, we provide here the first report that documents WFA's selective killing effects against oral cancer cells.

However, HGF-1 normal human gingival cells are a type of fibroblast cell lines and oral cancer cells arise from the epithelium. It was reported that some epithelial and fibroblast cells had different responses to various signaling molecules. For example, epithelial cells undergo growth arrest in response to TGF-β, whereas fibroblasts undergo morphological changes, and proliferate (Wilkes et al., 2003). Therefore, the possibility of selective killing of WFA against oral cancer cells warrants further detailed investigation. In a follow-up study we will compare oral cancer cells with normal epithelial cells using a similar experimental design.

### WFA induces apoptosis in cancer cells

In the current study, WFA induced apoptosis in Ca9-22 oral cancer cells associated with subG1 accumulation and caspase activation. This is consistent with the findings that WFA induced apoptosis in CaSki cervical cancer cells (Munagala et al., 2011) and WFA induced apoptosis in MDA-MB-231 and MCF-7 breast cancer cells (Hahm and Singh, 2013). In the current study (Figure 4E), the cleaved caspases (3, 8, and 9) and cleaved PARP showed high cleavage (high apoptosis) in 1 and 2 μM of WFA, but showed less cleavage (less apoptosis) in 3 μM of WFA. Similarly, other drugs have related findings. For example, etoposide induced more cleavage of PARP at 2 and 4 days, but declined at day 6 in human non-small cell lung cancer cells (H1437) (Chiu et al., 2005). Ganciclovir induced more cleavage of PARP, caspases 3/9, and cytochrome *c* at 1 or 2 days, but declined at 3 days in human non-small cell lung cancer cells (CL-1) (Chiu et al., 2002). It is possible that higher dose treatment or longer exposure may lead to more cell death and fail to proportionally cleave apoptotic proteins. Furthermore, caspase-1 may mediate apoptosis (Bergsbaken et al., 2009; Miao et al., 2011; Sollberger et al., 2014) and it warrants further investigation for the role of caspase-1 in WFA induced apoptosis.

In addition to the induction of subG1 accumulation, WFA induces G2/M arrest and a mitotic catastrophe in prostate cancer cells in terms of cell cycle analysis and western blotting for several G2/M arresting proteins (Roy et al., 2013). WFA also induces G2/M arrest in gastric cancer cells (Kim et al., 2017), osteosarcoma cells (Lv and Wang, 2015), and breast cancer cells (Zhang et al., 2011). Similar to the current study, WFA induced G2/M arrest in Ca9-22 cells at lower concentrations (1 and 2 μM) but did not present at higher concentration (3 μM) (Figure 2B) where subG1 population (apoptosis) was gradually increased.

### WFA induces DNA damage in cancer cells

Drug-induced ROS generation is associated with DNA damage (Yang et al., 2012; Chiu et al., 2013; Chen et al., 2016). Since WFA is known to induce ROS production (Lee et al., 2009; Li et al., 2017; Liu et al., 2017) (Figure 5), the DNA damage effect warrants for investigation. In the current study, we found that WFA treatment induced γH2AX expression in Ca9-22 oral cancer cells, which is consistent with the findings that WFA-induced γH2AX expression in MCF7 breast cancer cells, but not in normal human TIG-3 fibroblasts (Widodo et al., 2010). This DNA damaging ability was shown to be associated with apoptosis in several other cancer cell studies (Norbury and Zhivotovsky, 2004; Roos and Kaina, 2006).

### WFA-induced cell killing, apoptosis, cell cycle change, and DNA damage are medidated by oxidative stress in cancer cells

NAC is a common free radical scavenger. NAC pretreatment is reported to effectively diminish cellular ROS and to confirm the role of oxidative stress in drug treatment. Recently, several WFA-based cancer therapies were reported to be rescued by NAC pretreatment. For example, WFA induced apoptosis in human melanoma cells was accomplished by generating ROS (Mayola et al., 2011), and NAC pretreatment rescued ROS-induced damage. NAC also inhibited WFA-induced ROS production and caspase activation of human HL-60 myeloid leukemia cells (Malik et al., 2007).

Similarly, we found that NAC pretreatment rescued WFA-induced apoptosis in Ca9-22 oral cancer cells by subG1 accumulation, annexin V/PI, pan-caspases, and caspase signaling in western blotting. In our study, low concentration of WFA-induced G2/M arrest in Ca9-22 cells was rescued by NAC pretreatment. NAC pretreatment rescued WFA-induced oxidative stress in Ca9-22 cells by ROS and MMP analysis. NAC pretreatment also rescued γH2AX-based DNA damage in Ca9-22 cells as shown by the flow cytometry assay. This is the first evidence that NAC pretreatment protects WFA-treated oral cancer cells against oxidative stress.

## Conclusion

WFA selectively killed oral cancer cells with less toxic effects to normal oral cells. WFA also induced apoptosis, oxidative stress, and DNA damage, which ultimately inhibited the proliferation of oral cancer cells.

## Author contributions

JL, HW, and YC carried out the experiments. JT and HH analyzed the data. HC, RL, and CY conceived and designed the study. HC and CY wrote and revised the manuscript.

### Conflict of interest statement

The authors declare that the research was conducted in the absence of any commercial or financial relationships that could be construed as a potential conflict of interest.
